# Modelling Arterial Pressure Waveforms Using Gaussian Functions and Two-Stage Particle Swarm Optimizer

**DOI:** 10.1155/2014/923260

**Published:** 2014-05-20

**Authors:** Chengyu Liu, Tao Zhuang, Lina Zhao, Faliang Chang, Changchun Liu, Shoushui Wei, Qiqiang Li, Dingchang Zheng

**Affiliations:** ^1^School of Control Science and Engineering, Shandong University, Jinan 250061, China; ^2^School of Information Science and Engineering, Shandong University, Jinan 250100, China; ^3^Institute of Cellular Medicine, Newcastle University, Newcastle upon Tyne NE2 4HH, UK; ^4^National Laboratory of Pattern Recognition, Institute of Automation, Chinese Academy of Sciences, Beijing 100190, China

## Abstract

Changes of arterial pressure waveform characteristics have been accepted as risk indicators of cardiovascular diseases. Waveform modelling using Gaussian functions has been used to decompose arterial pressure pulses into different numbers of subwaves and hence quantify waveform characteristics. However, the fitting accuracy and computation efficiency of current modelling approaches need to be improved. This study aimed to develop a novel two-stage particle swarm optimizer (TSPSO) to determine optimal parameters of Gaussian functions. The evaluation was performed on carotid and radial artery pressure waveforms (CAPW and RAPW) which were simultaneously recorded from twenty normal volunteers. The fitting accuracy and calculation efficiency of our TSPSO were compared with three published optimization methods: the Nelder-Mead, the modified PSO (MPSO), and the dynamic multiswarm particle swarm optimizer (DMS-PSO). The results showed that TSPSO achieved the best fitting accuracy with a mean absolute error (MAE) of 1.1% for CAPW and 1.0% for RAPW, in comparison with 4.2% and 4.1% for Nelder-Mead, 2.0% and 1.9% for MPSO, and 1.2% and 1.1% for DMS-PSO. In addition, to achieve target MAE of 2.0%, the computation time of TSPSO was only 1.5 s, which was only 20% and 30% of that for MPSO and DMS-PSO, respectively.

## 1. Introduction

Changes of arterial pressure waveform characteristics have been accepted as risk indicators of cardiovascular diseases [1–3]. It is traditionally accepted that arterial pressure waveform contains both forward and backward components [4, 5]. However, the underlying physiological mechanisms of these components have not been fully understood. The backward component of arterial pressure waveform could be introduced by significant decrease in diameter and the change of elasticity at the juncture between thoracic and abdominal aorta or between abdominal aorta and common iliac arteries [5]. However, other studies suggested that there are no precise reflection sites in the arterial system [6]. These controversial conclusions could be caused by inaccurate identification of arterial pulse characteristics. Therefore, determining the arterial pressure waveform characteristics accurately is of clinical importance. It could provide better understanding of the pathophysiology of cardiovascular disease and identify risk indicators in patients with hypertension, chronic kidney disease, arteriosclerosis, or peripheral vascular diseases [7–10].

The common methods to assess arterial pressure waveform characteristics include mathematic model analysis, derivative methods, and wave intensity analysis [8, 11, 12]. Wave separation analysis, using a mathematic model, could acquire the forward and backward components provided that both aortic pressure and flow waveforms are given [13]. This model approach has been considered as a gold standard to assess wave reflection [8]. However, recording both aortic pressure and flow waveforms is practically difficult and hence its application is limited. Other researchers used derivative methods, including the first [14], second [15], or third derivatives [16] of the arterial pressure waveform, augmentation index [17], and wave intensity analysis [12], to explore the different characteristics of arterial pressure waveforms. However, these techniques are highly susceptible to noise.

To obtain a complete feature of arterial pressure waveform, pulse decomposition analysis has been used to decompose the pressure waveform into several independent subwaves. Different mathematic functions, including the triangular [8], logarithmic normal [18, 19], and Gaussian functions [20–22], have been implemented. However, the pulse decomposition analysis needs to set up initial parameters. Traditionally, these initial parameters are acquired from the first or second derivative of arterial pressure waveforms, but the derivative methods are sensitive to noise. In our recent study [23], using particle swarm optimizer (PSO) algorithm, the initial parameters of Gaussian functions were not required, and the modelling results demonstrated that using Gaussian and PSO optimization could achieve accurate arterial pressure waveform fitting. Moreover, significant changes in arterial pressure waveform characteristics have been observed in heart failure patients in comparison with normal subjects [24], indicating the clinical significance of modelling arterial pressure waveforms using Gaussian functions.

However, using PSO algorithms to determine the optimal parameters of Gaussian functions is sometimes in a dilemma: algorithms with strong ability of global optimization usually have slow convergence speed on unimodal problems, whereas algorithms with fast convergence speed on unimodal problems often have poor performance in solving complex multimodal problems [25, 26]. In the case of the arterial pressure waveform modelling, the function to be optimized is multimodal, containing multiple local optima. In our published study [23], the fully informed particle swarm algorithm proposed in [27] was used. Although a reasonable fitting accuracy has been achieved, its computation time was too long, typically over 10 s to model a single pulse. It is therefore essential to develop a better PSO algorithm that could perform on multimodal problem efficiently.

The aim of the study was to develop a two-stage particle swarm optimizer (TSPSO) to determine the optimal parameters of the Gaussian functions for accurately and efficiently modelling arterial pressure waveforms. Its performance in terms of fitting accuracy and computation time was compared with three classical optimization methods: the Nelder-Mead [28], the modified PSO (MPSO) method [29], and the dynamic multiswarm particle swarm optimizer (DMS-PSO) [30].

## 2. Materials and Methods

### 2.1. Ethics Statement

Twenty normal volunteers (8 female and 12 male, mean age 51 years) were enrolled at Qilu Hospital of Shandong University. All volunteers gave their written informed consent to participate in the study and confirmed that they had not participated in any other “clinical trial” within the previous three months. The study obtained a full approval from the Clinical Ethics Committee of the Qilu Hospitals of Shandong University and all clinical investigations were conducted according to the principles expressed in the Declaration of Helsinki.

### 2.2. Data Collection

All volunteers had normal electrocardiogram (ECG), ultrasonic cardiogram (UCG), blood lipid, and glucose. Volunteers with severe organ damage or with psychiatric disorders were excluded. Basic clinical information including age, height, weight, body mass index, and heart rate was firstly obtained. Manual auscultatory systolic and diastolic blood pressure (SBP and DBP) were measured by an experienced operator at the beginning of signal recording. The mean arterial pressure (MAP) and pulse pressure (PP) were calculated using the classic formulas: MAP = DBP + (SBP − DBP)/3 and PP = SBP − DBP. The clinical information is briefly summarized in Table 1.

All measurements were undertaken in a quiet, temperature-controlled (25 ± 3°C) measurement room. Before the formal recordings, the volunteer lays supine on a measurement bed for 10 min to allow cardiovascular stabilization. Standard lead-II ECG, carotid artery pressure waveform (CAPW), and radial artery pressure waveform (RAPW) were then simultaneously and digitally recorded for 1 min using the Cardiovascular System Function Detecting Instrument (HUIYIRONGGONG Ltd., China) at a sample rate of 1000 Hz.

**Figure 1:**
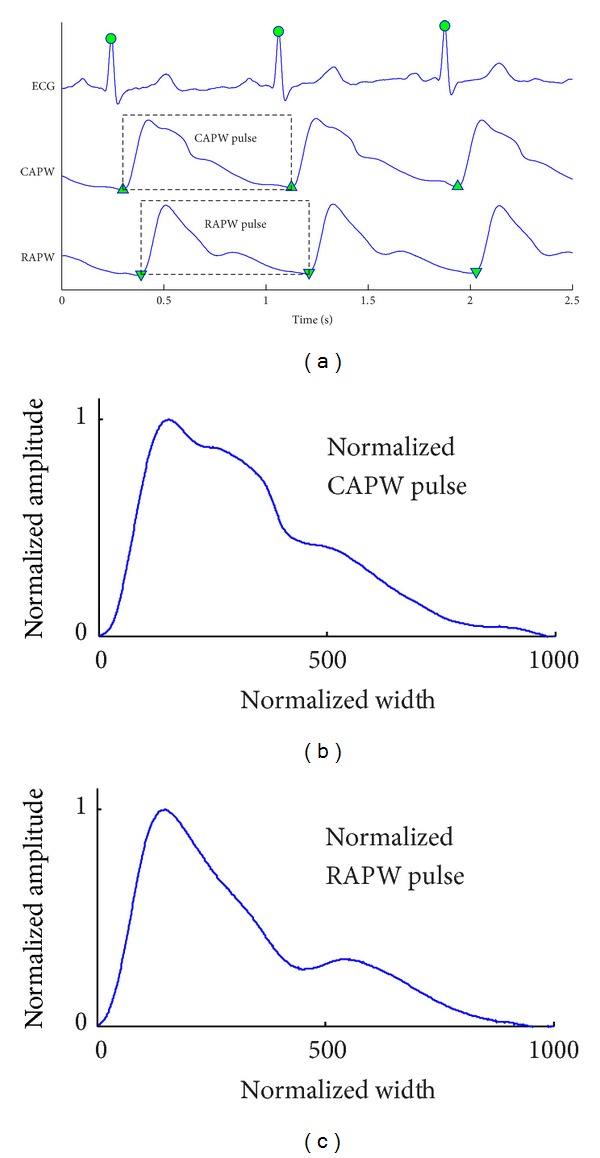
(a) One example of recorded ECG, carotid artery pressure waveform (CAPW), and radial artery pressure waveform (RAPW) signals. The detected R-wave peaks are denoted by “●”, and the starting points of CAPW and RAPW signals are denoted by “▲” and “▼,” respectively. (b) Normalized CAPW and (c) RAPW pulses with width up to 1000 points and the amplitude to unity between 0 and 1.

Offline analysis was performed by a custom designed computer program developed with MATLAB (version R2009a, MathWorks Inc., USA). First, the baseline (0–0.05 Hz) was removed for the ECG signal; the band-pass filter (0.05–35 Hz) was used for the CAPW and RAPW signals. Second, the R-wave peaks of the ECG were detected using the wavelet transform modulus maxima method [31]. Ectopic beats were identified and excluded [32]. After the location of R-wave peaks, their corresponding pulse feet (start of pulse) were identified [33]. The CAPW and RAPW signals were then segmented between the starting points of two consecutive pulses.  Figure 1 shows an example of the three signals with the features identified. Each pulse segment corresponded to one cardiac cycle and was normalized in width and amplitude, with the width up to 1000 points and the amplitude to unity between baseline and peak. The first 10 successive pulses without ectopic beats were used for subsequent waveform fitting analysis. Using 10 pulses ensured that the variation over a respiratory period was included.

### 2.3. Arterial Pulse Waveform Modelling

Our previous study [23] reported that both carotid and radial pulses could be accurately and reliably modelled using three positive Gaussian functions. The three Gaussian functions were denoted by *f*_1_(*n*), *f*_2_(*n*), and *f*_3_(*n*). Each Gaussian function *f*_*k*_(*n*)  (*k* = 1, 2, 3) had 1000 points (*n* = 1,2,…, 1000) and was determined by three parameters: waveform height *H*_*k*_, half-width *W*_*k*_, and the center position *C*_*k*_. The Gaussian functions are defined as follows:

(1) $$\begin{array}{c} f_{k}\left(n\right)=H_{k}\times \exp\left(-\frac{2{\left(n-C_{k}\right)}^{2}}{W_{k}^{2}}\right), \end{array}$$

where *C*_*k*_ satisfies the following condition: 1 < *C*_1_ < *C*_2_ < *C*_3_ < 1000.

After nine parameters *H*_*k*_, *W*_*k*_, and *C*_*k*_ were determined, the superimposed curve *f*(*n*, *x*) of the three Gaussian functions was regarded as the modelled curve for the original pulse *S*(*n*):

(2) $$\begin{array}{c} f\left(n,x\right)=\overset{3}{\underset{k=1}{\sum }}f_{k}\left(n\right), \end{array}$$

where *x* = [*H*_*k*_, *W*_*k*_, *C*_*k*_]  (*k* = 1,2, 3) was the parameter vector.

### 2.4. Two-Stage Particle Swarm Optimizer

Waveform fitting with a superposition of three Gaussians is essentially an optimization problem. The objective function is expressed as follows:

(3) $$\begin{array}{c} \mathrm{Min}\;F\left(x\right)=\overset{1000}{\underset{n=1}{\sum }}{\left[f\left(n,x\right)-S\left(n\right)\right]}^{2}, \end{array}$$

where *x* is the parameter vector to be optimized.

**Figure 2:**
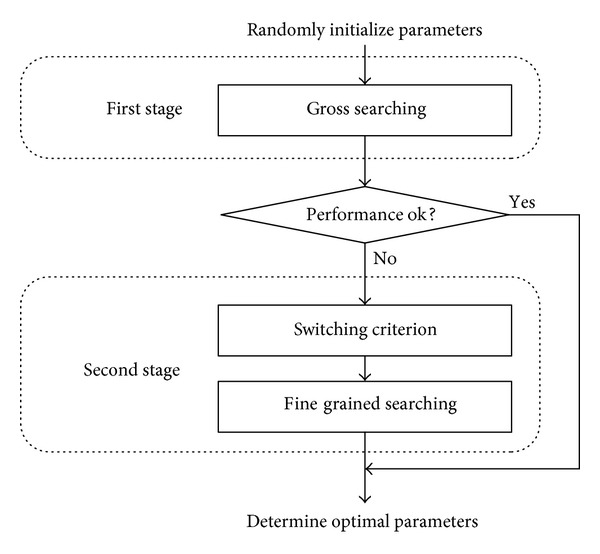
Searching mechanism of the two-stage particle swarm optimizer (TSPSO).

In this study, TSPSO was developed to solve the optimization problem in (3). As shown in Figure 2, it had three main components: a gross searching algorithm at the first stage and a switching criterion and a fine-grained searching algorithm at the second stage. According to the outcome of the gross searching, if its solutions were considerably improved, the search would stop at predefined maximum number of function evaluations (*FEs*), named as* max_FEs*, or predefined fitting accuracy. If stagnation occurred, it would then switch to a fine-grained searching algorithm automatically for better solution.

#### 2.4.1. Gross Searching Algorithm at the First Stage

A fully informed particle swarm was used for the gross searching algorithm due to its simplicity and good performance on simple optimization problems [27].

The dimension (*D*) of the search space was 9 since nine Gaussian parameters were used in the arterial pulse modelling. Let *P* denote the size of the swarm; each particle *i*  (1 ≤ *i* ≤ *P*) had the following attributes: its current velocity *V*_*i*_ = (*V*_*i*_^1^, *V*_*i*_^2^,…, *V*_*i*_^9^), its current position in the search space *X*_*i*_ = (*X*_*i*_^1^, *X*_*i*_^2^,…, *X*_*i*_^9^), and a personal best position *pbest*_*i*_ = (*pbest*_*i*_^1^, *pbest*_*i*_^2^,…, *pbest*_*i*_^9^). The swarm had best position discovered from the population in the search space *gbest* = (*gbest* ^1^, *gbest* ^2^,…, *gbest* ^9^). The velocity *V*_*i*_^*d*^ and position *X*_*i*_^*d*^ of the *d*th dimension of the *i*th particle were updated as follows:

(4) $$\begin{array}{c} V_{i}^{d}=\chi \left(V_{i}^{d}+\varphi_{1,i}^{d}\left({pbest}_{i}^{d}-X_{i}^{d}\right)+\varphi_{2,i}^{d}\left({gbest}_{i}^{d}-X_{i}^{d}\right)\right),\\ X_{i}^{d}=X_{i}^{d}+V_{i}^{d}, \end{array}$$

where 1 ≤ *i* ≤ *P*, 1 ≤ *d* ≤ 9, *χ* is the constraint coefficient, *φ*_1,*i*_^*d*^, *φ*_2,*i*_^*d*^ ~ *U*(0, *φ*/2), *U* is uniform distribution, and *φ* is a constant. The recommended values of *χ* = 0.73 and *φ* = 4.15 were used [27].

#### 2.4.2. Switching Criterion

In principle, TSPSO switches to the second stage if the gross searching algorithm could not improve the solutions further. In order to reduce the computation time, TSPSO switches only at points *k* = ⌊*mT*⌋, where *m* = 1,2,…, *h*,  *T* = *max*_*FEs*/*h*, and *h* is a positive integer. *T* is the interval between two adjacent switch-deciding points, and *h* is the number of possible switching times. For example, if *h* = 20, TSPSO only makes a maximum of 20 decisions on whether to switch. The computation time to make these decisions is negligible.

The switching criterion is as follows:

(5) if log10(gbestval((m−1)T))  −log10(gbestval(mT))<thr,then TSPSO  should  swith  to  the  second  stage,

where *thr* is the threshold used to compare the solution improvement speed with the value more than 0 and *gbest* *v* *a* *l*(*p*) is the output of the objective function in (3) (i.e., *gbest* *v* *a* *l*(*p*) = *F*(*gbest*(*p*))) given that *gbest*(*p*) is the best solution determined by the gross searching algorithm after* p FEs*. Parameter *thr* controls the strictness of the switching criterion. The smaller the value of* thr* is, the harder the criterion is to be satisfied. An empirical value of *thr* = 0.03 could effectively achieve waveform fitting.

Equation (5) is equivalent to the following equation:

(6) $$\begin{array}{c} \frac{gbestval\left(\left(m-1\right)T\right)}{gbestval\left(mT\right)}<10^{thr}. \end{array}$$

From (6), it can be seen that if the current solution has not been improved very much after *k* = (*m* − 1)*T*, at the point *k* = *mT*, the value of *gbest* *v* *a* *l*  ((*m* − 1)*T*)/*gbest* *v* *a* *l*  (*mT*) is close to 1, which satisfies the switching criterion in (5); TSPSO switches to the second stage at the point *k* = *mT*. If the current solution still had a considerable improvement, TSPSO would not switch.

#### 2.4.3. Fine-Grained Searching Algorithm at the Second Stage

A fine-grained searching algorithm was designed at the second stage of TSPSO. After the first stage, the swarm has already converged to a good *gbest* and this *gbest* was used as the initial population best position for the second stage.

Suppose that the output at the first stage is *gbest* = (*gbest* ^1^, *gbest* ^2^,…, *gbest* ^9^); since *gbest* is usually close to the global optimum on some dimensions, a dimension by dimension strategy was used for the fine-grained searching. When it optimized the *d*th dimension of *gbest*, a one-dimensional swarm of size *P* was randomly initialized as *X*_*i*_  (1 ≤ *i* ≤ *P*). *b*(*d*, *X*_*i*_) = (*gbest* ^1^, *gbest* ^2^,…, *gbest* ^*d*−1^, *X*_*i*_, *gbest* ^*d*+1^,…, *gbest* ^9^) was then defined, and *F*(*b*(*d*, *X*_*i*_)) was used as the fitness of particle *i*. After the initialization, this one-dimensional swarm optimized the *d*th dimension of *gbest* using PSO with inertia weight update scheme as follows [34]:

(7) Vid=wVid+c1rand1id[pbestid−Xid]+c2rand2id[gbestd−Xid],Xid=Xid+Vid,

where *c*_1_ and *c*_2_ are the acceleration constants reflecting the weighting of stochastic acceleration terms that pull each particle toward *pbest* and *gbest* positions, respectively; rand1_*i*_^*d*^ and rand2_*i*_^*d*^ are two random numbers in the range of [0, 1]; and *w* is the inertia weight. The maximum number of* FEs* for this one-dimensional swarm optimization was set to *oneD*_*FEs*. After the completion of optimization in this *d*th dimension, the population best position of this swarm was used to improve its *gbest*, which was reinitialized to optimize the (*d* + 1)th dimension of *gbest*. After the swarm optimized all the 9 dimensions of *gbest*, this process was repeated from the first dimension until the predefined target *max*_*FEs* or the fitting accuracy was reached.

### 2.5. Comparison with Published Optimization Methods

Our TSPSO was compared with three published optimization algorithms: Nelder-Mead [28], MPSO [29], and DMS-PSO [30]. Nelder-Mead method uses a direct search algorithm without computing gradients. It has been widely used with good performance on solving local optimization problems. MPSO method is based on a typical one-stage global PSO algorithm. DMS-PSO is a local version of PSO with a dynamic and randomized neighborhood topology, as well as using small subswarms' size. For the implementation of DMS-PSO, the particle population size was set as 9, with subswarm size *m* = 3 and regrouping period *R* = 20.

### 2.6. Waveform Fitting Assessment

The performance of waveform fitting was evaluated in terms of fitting accuracy and computation time for a required fitting accuracy. The fitting accuracy of the four algorithms (Nelder-Mead, MPSO, DMS-PSO, and TSPSO) was assessed by the mean absolute error (MAE), which is expressed as follows:

(8) $$\begin{array}{c} \text{MAE}=\frac{\sum_{n=1}^{N}\left|f\left(n,x\right)-S\left(n\right)\right|}{N}\times 100\%, \end{array}$$

where *f*(*n*, *x*) is the fitting result using three Gaussian functions, *S*(*n*) is the original pulse, and *N* is the total number of normalized width and is 1000 in this study.

All the algorithms could be stopped by a predefined target MAE or* max_FEs*. If target MAE was 0, the algorithms were terminated by* max_FEs*, and the fitting accuracy could be compared between methods. If a specific nonzero MAE was selected to stop the algorithms, the computation time was obtained and compared. The assessment of waveform fitting was performed using MATLAB software (version R2009a, MathWorks Inc., USA) on Windows XP platform (CPU: Intel Core i5, 2.66 GHz).

### 2.7. Statistical Analysis

The average MAE and computation time for each volunteer were firstly calculated from the 10 beats used for waveform fitting. The overall mean and standard deviation (SD) of MAE were then obtained across the 20 volunteers. Analysis of variance (ANOVA) and post hoc multiple comparison were performed to investigate the effect of using different methods on MAE and computation time. All statistical analyses were performed using the Statistical Package for Social Sciences (v. 19, SPSS Inc., Chicago, IL, USA) and a value of *P* < 0.05 was considered statistically significant.

## 3. Results

### 3.1. Fitting Accuracy

**Figure 3:**
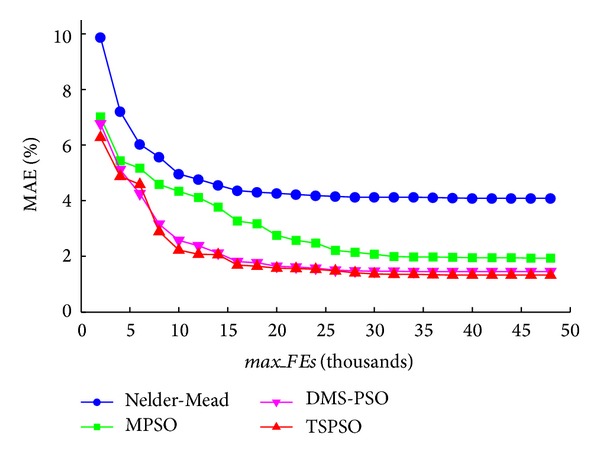
Mean absolute error (MAE) changes with different maximum numbers of function evaluations* max_FEs*. The results of the four methods (Nelder-Mead, MPSO, DMS-PSO, and TSPSO) are shown.

Figure 3 shows the changes of waveform fitting accuracy with increasing* max_FEs* from one typical arterial pulse. For all four methods, it can be seen that MAE decreased with increased* max_FEs* and reached a stable value with* max_FEs*  ≥ 30000. The smallest MAE values were achieved by the DMS-PSO and TSPSO methods.

**Figure 4:**
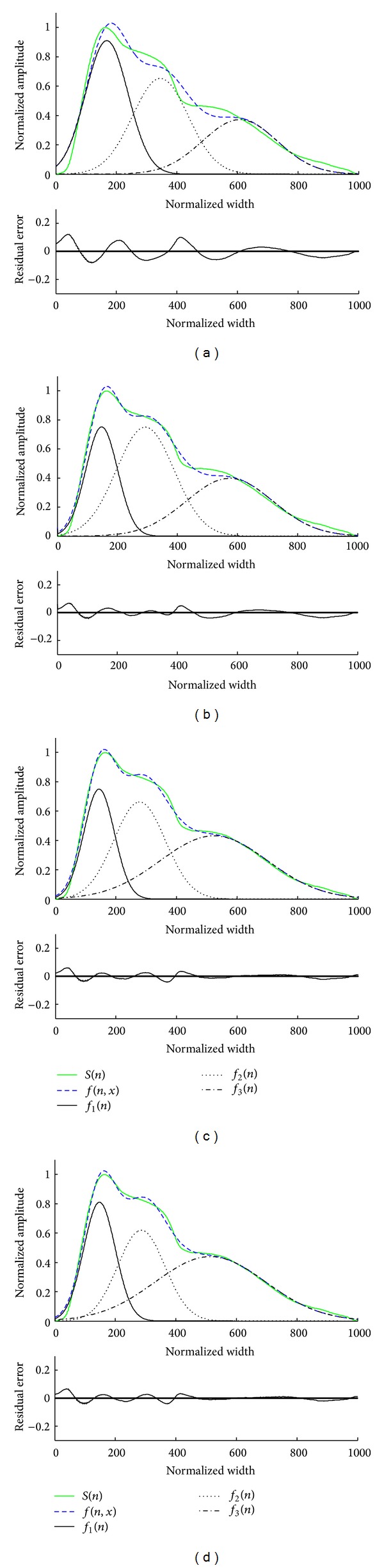
Waveform fitting of a normalized pulse using four different methods. (a) Nelder-Mead, (b) MPSO, (c) DMS-PSO, and (d) TSPSO. In each subfigure, the upper panel shows the original normalized pulse *S*(*n*), the fitting curve using three Gaussian functions *f*(*n*, *x*), and the corresponding three Gaussian functions *f*_1_(*n*), *f*_2_(*n*), and *f*_3_(*n*) from left to right in turn. The bottom panel shows the corresponding residual error between the original and fitted waveforms.

Figure 4 shows a waveform fitting example using the four methods. The* max_FEs* of 30000 was used here since each method could achieve stable MAE beyond this level. The MAEs were 4.2%, 2.0%, 1.5%, and 1.4%, respectively, for the Nelder-Mead, MPSO, DMS-PSO, and TSPSO methods. From this example pulse, it can be seen that DMS-PSO and TSPSO could model the arterial pulse better than Nelder-Mead and MPSO.

**Figure 5:**
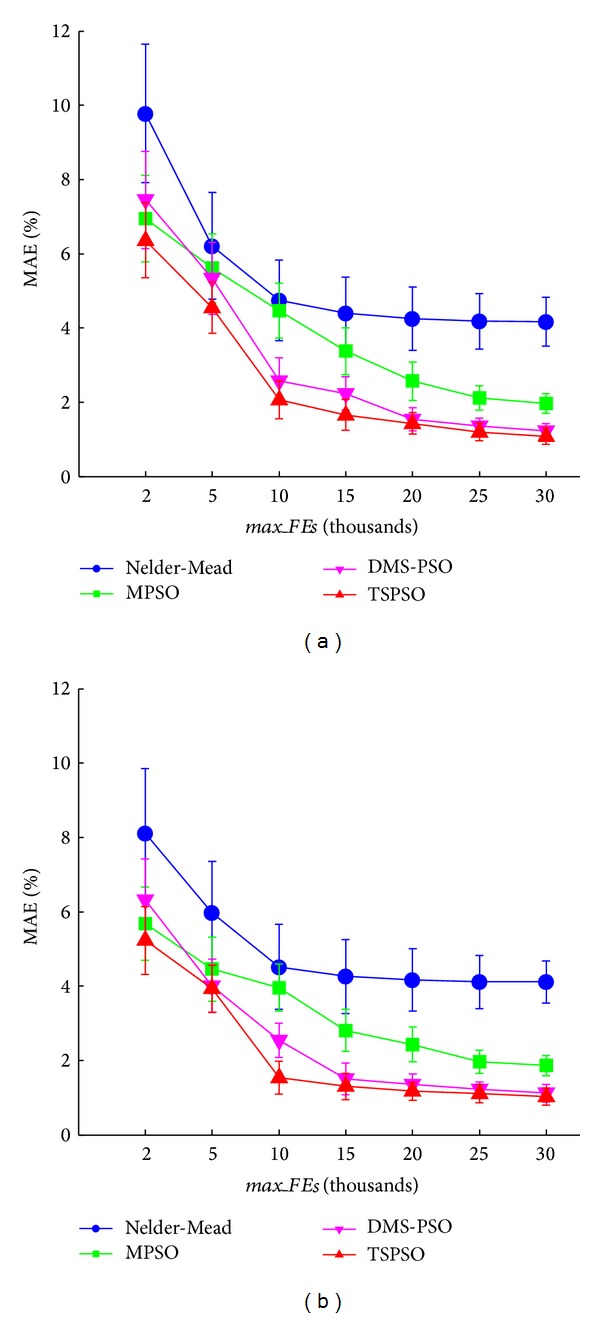
Means and standard deviations (SDs) of MAE for the four methods (Nelder-Mead, MPSO, DMS-PSO, and TSPSO). The results from 20 volunteers are given at different maximum numbers of function evaluations* max_FEs* levels (2000, 5000, 10000, 15000, 20000, 25000, and 30000), (a) for CAPW signal and (b) for RAPW signal.

Figure 5 and Table 2 give the overall mean and SD of MAE with different* max*_*FEs* for both CAPW and RAPW signals. TSPSO achieved the best fitting accuracy with the smallest MAEs, and Nelder-Mead had the worst fitting accuracy with the largest MAEs. In comparison with Nelder-Mead and MPSO methods, TSPSO achieved significantly lower MAEs at all* max*_*FEs* levels (all *P* < 0.05, except for the comparison with MPSO at* max*_*FEs* = 2000). TSPSO also achieved significantly lower MAEs (*P* < 0.05) at* max*_*FEs* ≤ 15000 when compared with DMS-PSO. The best fitting results were achieved from TSPSO method with* max*_*FEs* = 30000 and its corresponding MAE values were 1.1 ± 0.2% for CAPW signal and 1.0 ± 0.2% for RAPW signal.

### 3.2. Computation Time

**Figure 6:**
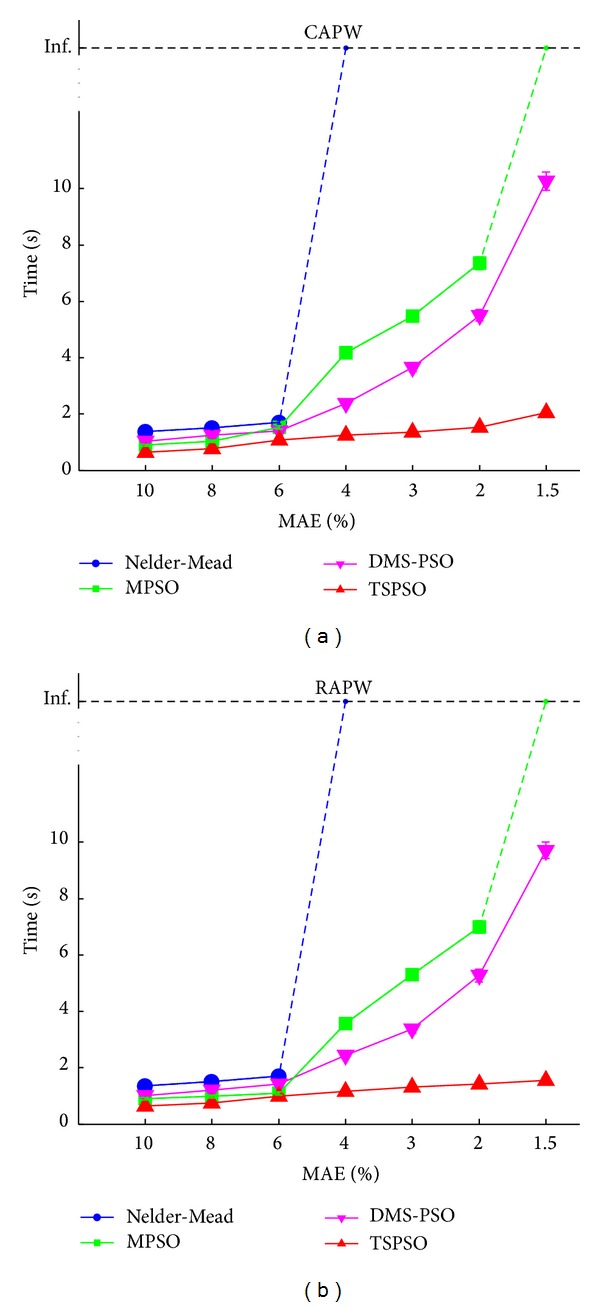
Means and standard deviations (SDs) of the computation time for the four methods (Nelder-Mead, MPSO, DMS-PSO, and TSPSO). The results from 20 volunteers are given at different mean absolute error (MAE) levels (10%, 8%, 6%, 4%, 3%, 2%, and 1.5%), (a) for CAPW signal and (b) for RAPW signal. The SDs of computation time at all MAE levels are relatively small. “Inf.” indicates that the target MAE level can not be achieved even with extremely large maximum number of function evaluations* max_FEs* (100000).

Figure 6 and Table 3 show the computation time required to achieve different predefined target MAE levels. To achieve target MAE ≥ 6%, although all four methods completed the waveform fitting within 2 s, TSPSO required significantly less time than Nelder-Mead, MPSO, and DMS-PSO methods (all *P* < 0.05). To achieve relatively accurate fittings (target MAE ≤ 4%), the computation time differences between TSPSO and the other three methods became large, and Nelder-Mead could not achieve the target. To further increase the fitting accuracy, the computation times of MPSO and DMS-PSO increased obviously whereas TSPSO increased slowly. To achieve the MAE of 2.0%, the computation time of TSPSO was 1.5 s, which was only 20% of that for MPSO (for CAPW, 1.5 versus 7.4 s; for RAPW, 1.4 versus 7.0 s) and was only 30% of that for DMS-PSO (for CAPW, 1.5 versus 5.5 s; for RAPW, 1.4 versus 5.3 s).

In addition, only DMS-PSO and TSPSO achieved the mean MAE less than 1.5%. The computation time of TSPSO was 2.0 s and 1.6 s, respectively, for CAPW and RAPW signals, which were significantly lower (*P* < 0.05) than those for DMS-PSO (for CAPW, 2.0 versus 10.3 s; for RAPW, 1.6 versus 9.7 s).

## 4. Discussion and Conclusion

The major finding of this study was that, using the proposed TSPSO algorithm and three Gaussians, both CAPW and RAPW signals could be accurately modelled. To the best of our knowledge, TSPSO was used for the first time to fit arterial pressure waveform when using Gaussian functions. To model the arterial pressure pulses, some published studies separated pulse waveforms into two components by a triangular wave of duration equal to the ejection time [8] or using a two-pulse synthesis model [35]. However, because the arterial pulse waveforms are often complicated with three or more components, other studies used three subwaves [36], four subwaves [22], and even five subwaves [5, 18, 19] to model the pulses. In terms of fitting accuracy, Rubins reported that the residual error between the original and modelled pulse waveforms did not exceed 10% [22]. Huotari's study provided only some examples with an average maximum residual error of 4% [18, 19]. Xu et al. used an adaptive number (four or five) of subwaves and achieved the fitting error less than 2% [20, 21]. However, the above studies used derivatives methods that were to set up the initial parameters, which were sensitive to noise, and there is also no physiological explanation of why four or five functions are needed. Our proposed TSPSO method determined the optimal Gaussian parameters without initial parameters and achieved the fitting error less than 1.5% only using three Gaussian functions, which was the best result among all the published studies. In addition, the comparison with some classical PSO algorithms was also performed in this study. Overall, TSPSO algorithm achieved significantly better accuracy than Nelder-Mead and MPSO methods at most* max*_*FEs* levels and also significantly better accuracy than DM-PSO method at small* max*_*FEs* levels.

The second major finding was that it was possible to efficiently model both CAPW and RAPW signals using TSPSO. To the best of our knowledge, this is the first study to compare the computation efficiency between different algorithms. Nelder-Mead method had poor fitting accuracy, which limited its application. Although both DMS-PSO and TSPSO could achieve highly accurate fitting with MAE ≤ 1.5%, the computation time of TSPSO was significantly less than DMS-PSO. In comparison with MPSO and DMS-PSO methods, TSPSO is a two-stage PSO and automatically switches to a fine-grained searching stage and hence reduces the computation time. Taking the best achievable fitting accuracy into account, TSPSO was concluded as the most efficient method for modelling arterial pulses with the shortest computation time.

In addition, another multistage optimization algorithm, named tree search dynamic multiswarm particle swarm optimizer (TS-DMS-PSO) [37], has been reported. It also performs gross searching firstly before refining the result using a highly accurate fitness function. There are two main differences between the TS-DMS-PSO and TSPSO: (1) TS-DMS-PSO is a local PSO version with the velocity of each particle modified from its personal best and the best performance achieved so far within its neighborhood. Our TSPSO is a global approach, learning from the personal best and the best position achieved so far by the whole population. (2) TS-DMS-PSO uses the same searching strategy at different stages. At each stage, it reinitializes the searching step and the searching range. However, TSPSO has different searching strategies for the two stages (gross and fine-grained searching stages). At the gross searching stage, TSPSO uses the common fully informed particle swarm method for all dimensions. After the first stage, it switches to the fine-grained searching stage, where PSO with inertia weight update scheme is used for each dimension. For the application of modelling the arterial pulses, the advantage of using TSPSO is that, after the gross searching stage, the three Gaussian functions could be optimized through single dimension searching strategy, resulting in significantly shorter computation time.

It is also worth noting that TSPSO could achieve similar fitting accuracy and effectiveness to model both CAPW and RAPW, confirming that this method can be used for the waveform analysis from different pulse sites (carotid artery and radial artery).

Currently, the physiological mechanism of wave reflection is still controversial. It has been reported that early wave reflection shifts proximally toward the heart with aging [38]. On the contrary, a distal shift of reflection site has been observed by Mitchell et al. [9], but others reported that this distal shift was only observed from subjects older than 65 years [7]. These controversial conclusions are partially due to the different methods used to identify the waveform characteristics [39]. Another explanation could be that those methods are not accurate enough to reconstruct the pressure waveforms [4]. Our proposed TSPSO method with three Gaussian functions provides an alternative tool to quantify the different components of arterial pressure waveform. And validating the clinical efficiency of TSPSO method would be our future work.

In summary, it has been demonstrated that TSPSO with three Gaussian functions is a promising pulse decomposition analysis method to model arterial pressure waveforms. Its accurate waveform fitting and short computation time provide great confidence for identifying arterial pressure waveform characteristics and hence provide better understanding of their underlying physiological mechanisms.

## Figures and Tables

**Table 1:** Summary of clinical characteristics from 20 volunteers.

Clinical characteristic	Value
Age, year	51 ± 11
Height, cm	171 ± 9
Weight, kg	69 ± 8
Body mass index, kg/m^2^	23 ± 3
Heart rate, beats/min	71 ± 8
Brachial SBP, mmHg	118 ± 12
Brachial DBP, mmHg	71 ± 10
Brachial MAP, mmHg	87 ± 9
Brachial PP, mmHg	47 ± 11

SBP: systolic blood pressure, DBP: diastolic blood pressure, MBP: mean arterial pressure, PP: pulse pressure.

Data are expressed as mean ± standard deviation (SD).

**Table 2:** Statistical results of MAE from 20 volunteers with different *max_FEs* values for the four methods.

*max_FEs *	CAPW (%)				RAPW (%)			
	Nelder-Mead	MPSO	DMS-PSO	TSPSO	Nelder-Mead	MPSO	DMS-PSO	TSPSO
2000	9.8 ± 1.9*	7.0 ± 1.2	7.5 ± 1.3*	6.4 ± 1.0	8.1 ± 1.7*	5.7 ± 1.0	6.3 ± 1.1*	5.2 ± 0.9
5000	6.2 ± 1.4*	5.6 ± 0.9*	5.3 ± 1.0*	4.6 ± 0.7	6.0 ± 1.4*	4.5 ± 0.9*	4.0 ± 0.7	3.9 ± 0.6
10000	4.7 ± 1.1*	4.5 ± 0.7*	2.6 ± 0.6*	2.1 ± 0.5	4.5 ± 1.2*	4.0 ± 0.6*	2.5 ± 0.5*	1.5 ± 0.4
15000	4.4 ± 1.0*	3.4 ± 0.6*	2.2 ± 0.5*	1.7 ± 0.4	4.3 ± 1.0*	2.8 ± 0.6*	1.5 ± 0.4	1.3 ± 0.4
20000	4.3 ± 0.9*	2.6 ± 0.5*	1.5 ± 0.3	1.4 ± 0.3	4.2 ± 0.8*	2.4 ± 0.5*	1.4 ± 0.3*	1.2 ± 0.2
25000	4.2 ± 0.8*	2.1 ± 0.3*	1.3 ± 0.2	1.2 ± 0.2	4.1 ± 0.7*	2.0 ± 0.3*	1.2 ± 0.2	1.1 ± 0.2
30000	4.2 ± 0.7*	2.0 ± 0.3*	1.2 ± 0.2	1.1 ± 0.2	4.1 ± 0.6*	1.9 ± 0.3*	1.1 ± 0.2	1.0 ± 0.2

Data are expressed as mean ± standard deviation (SD). MAE: mean absolute error; *max_FEs*: maximum number of function evaluations; CAPW: carotid artery pressure waveforms; RAPW: radial artery pressure waveforms.

*There is statistically significant difference when compared with TSPSO (*P* < 0.05).

**Table 3:** Statistical results of the computation time from 20 volunteers with different target MAEs for the four methods.

MAE (%)	CAPW (s)				RAPW (s)			
	Nelder-Mead	MPSO	DMS-PSO	TSPSO	Nelder-Mead	MPSO	DMS-PSO	TSPSO
10	1.4 ± 0.05*	0.9 ± 0.04*	1.0 ± 0.03*	0.6 ± 0.02	1.4 ± 0.04*	0.9 ± 0.04*	1.0 ± 0.04*	0.7 ± 0.02
8	1.5 ± 0.06*	1.0 ± 0.05*	1.3 ± 0.05*	0.8 ± 0.03	1.5 ± 0.06*	1.0 ± 0.05*	1.2 ± 0.04*	0.7 ± 0.04
6	1.7 ± 0.06*	1.5 ± 0.07*	1.4 ± 0.06*	1.1 ± 0.03	1.7 ± 0.07*	1.1 ± 0.05*	1.4 ± 0.05*	1.0 ± 0.04
4	Inf.	4.2 ± 0.12*	2.4 ± 0.09*	1.3 ± 0.06	Inf.	3.6 ± 0.12*	2.4 ± 0.10*	1.2 ± 0.05
3	Inf.	5.5 ± 0.19*	3.7 ± 0.16*	1.4 ± 0.07	Inf.	5.3 ± 0.16*	3.4 ± 0.16*	1.3 ± 0.06
2	Inf.	7.4 ± 0.23*	5.5 ± 0.21*	1.5 ± 0.07	Inf.	7.0 ± 0.21*	5.3 ± 0.23*	1.4 ± 0.08
1.5	Inf.	Inf.	10.3 ± 0.32*	2.0 ± 0.10	Inf.	Inf.	9.7 ± 0.29*	1.6 ± 0.09

Data are expressed as mean ± standard deviation (SD) or as symbol “Inf.” “Inf.” indicates that the method could not achieve the target MAE even with the extremely large *max_FEs* (100000). MAE: mean absolute error; CAPW: carotid artery pressure waveforms; RAPW: radial artery pressure waveforms.

*There is statistically significant difference when compared with TSPSO (*P* < 0.05).
